# Mimicking the Mechanical Properties of Aortic Tissue with Pattern-Embedded 3D Printing for a Realistic Phantom

**DOI:** 10.3390/ma13215042

**Published:** 2020-11-09

**Authors:** Jaeyoung Kwon, Junhyeok Ock, Namkug Kim

**Affiliations:** 1 Department of Biomedical Engineering, Asan Medical Center, University of Ulsan College of Medicine, 388-1 Pungnap2-dong, Songpa-gu, Seoul 138-828, Korea; nisroeld2@gmail.com (J.K.); jhock8755@gmail.com (J.O.); 2 Department of Convergence Medicine, Asan Medical Institute of Convergence Science and Technology, Asan Medical Center, University of Ulsan College of Medicine, 388-1 Pungnap2-dong, Songpa-gu, Seoul 138-828, Korea

**Keywords:** 3D printing, aorta, multi-materials, pattern embedding, tensile testing

## Abstract

3D printing technology has been extensively applied in the medical field, but the ability to replicate tissues that experience significant loads and undergo substantial deformation, such as the aorta, remains elusive. Therefore, this study proposed a method to imitate the mechanical characteristics of the aortic wall by 3D printing embedded patterns and combining two materials with different physical properties. First, we determined the mechanical properties of the selected base materials (Agilus and Dragonskin 30) and pattern materials (VeroCyan and TPU 95A) and performed tensile testing. Three patterns were designed and embedded in printed Agilus–VeroCyan and Dragonskin 30–TPU 95A specimens. Tensile tests were then performed on the printed specimens, and the stress-strain curves were evaluated. The samples with one of the two tested orthotropic patterns exceeded the tensile strength and strain properties of a human aorta. Specifically, a tensile strength of 2.15 ± 0.15 MPa and strain at breaking of 3.18 ± 0.05 mm/mm were measured in the study; the human aorta is considered to have tensile strength and strain at breaking of 2.0–3.0 MPa and 2.0–2.3 mm/mm, respectively. These findings indicate the potential for developing more representative aortic phantoms based on the approach in this study.

## 1. Introduction

Due to its ability to provide materials that are specific to their intended purposes, 3D printing technology has been widely utilized in the medical field [1,2,3] for applications such as surgical guides, implants, and scaffolds for cell culture that necessitate biocompatible materials [4,5,6]. For example, a 3D printed surgical guide is designed to support the patient-specific planning of surgeries, decrease surgery time, and minimize resection. Furthermore, 3D printed metallic implants are used for hip joints and maxilla [7,8]. In addition, 3D printing can fabricate customized medical and surgical tools with mechanically and chemically stable materials [9,10]. Biocompatible materials, including silicone, collagen, gelatin, and bio-inks stacked inside gelatin, are also under development [3,11]. Moreover, 3D printed educational models, simulators, and patient-specific phantoms can be produced based on medical images because the technology can replicate detailed anatomical structures [12,13,14]. In particular, flow experiments using 3D printed blood vessels or valve phantoms can provide an environment similar to the human body for the development of medical imaging protocols and the verification of diagnostic methods [15,16,17].

However, the elasticity and elongation of printed materials may be insufficient when the functional movement or the forces applied to the structure are significant, as is the case for the heart and the aorta. Wang et al. sought to overcome these limitations by using TangoPlus as a base material and embedding VeroBlackPlus in a sinusoidal and double helix manner [18,19]. Although the inherent differences in the mechanical behaviors of soft tissues and polymer could be decreased, the polymer did not demonstrate the ability to perform as well as human tissues under complex loading. Bandyopadhyay et al. reviewed multi-material additive manufacturing (AM) processes [20] and discussed the advantages and disadvantages of AM multi-material structures. Further, they highlighted the scope of 3D printed polymer-based, metal-metal, and metal-ceramic applications. The cases evaluated were primarily hard implants, with no discussion of soft tissue research. Yuan et al. proposed a new layout design strategy for 3D printed multi-material composites that incorporated thermally reactive shape memory polymer and flexible elastomers [21]. They advocated that the customization of shape memory properties could be applied to various research fields, including 4D printing, metamaterials, and biomimetic structures. Bass et al. explained the effects of the orientation of photopolymers containing various concentrations of elastomeric materials on their mechanical behaviors [22]. Waran et al. created a neurosurgical model by mimicking tissues using a multi-material 3D printer [23]. However, imitating highly deformable human tissues remains challenging.

Several studies have also investigated producing metamaterials by 3D printing. Wu et al. reviewed the mechanical design of 2D and 3D chiral metamaterials and investigated their mechanical behaviors and deformation mechanisms by applying the equilibrium principle, strain energy analysis, micropolar elasticity, and homogenization theories [24]. They introduced industrial applications of these chiral mechanical metamaterials, such as smart deployable antennas with morphing airfoils and reconfigurable structures, as well as auxetic stents for biomedical applications. Jiang et al. proposed a new hybrid chiral mechanical metamaterial that combined two basic deformation mechanisms for auxetic open cell metamaterials, i.e., re-entrant angle and chirality [25]. They suggested that this mechanism could be applied to drug delivery and develop new multifunctional smart composites, sensors, and actuators that respond to external loads or environmental conditions. Surjadi et al. introduced the fundamental science theory behind novel metamaterials and potential engineering applications beyond manufacturing techniques and mechanics, such as recent mechanical and functional applications [26]. Metamaterials can overcome the limitations of materials’ traditional mechanical behaviors, but previous studies have not addressed replicating human tissues.

**Figure 1:**
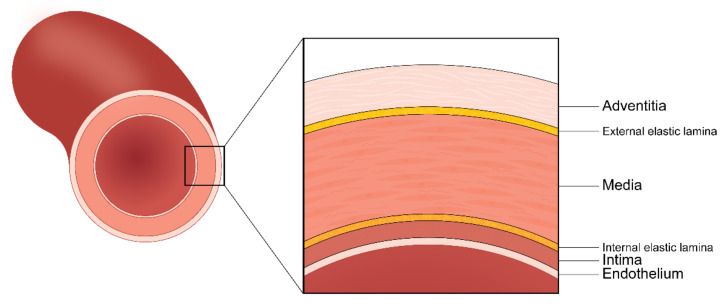
Multi-layered structure of a typical aortic wall.

**Figure 2:**
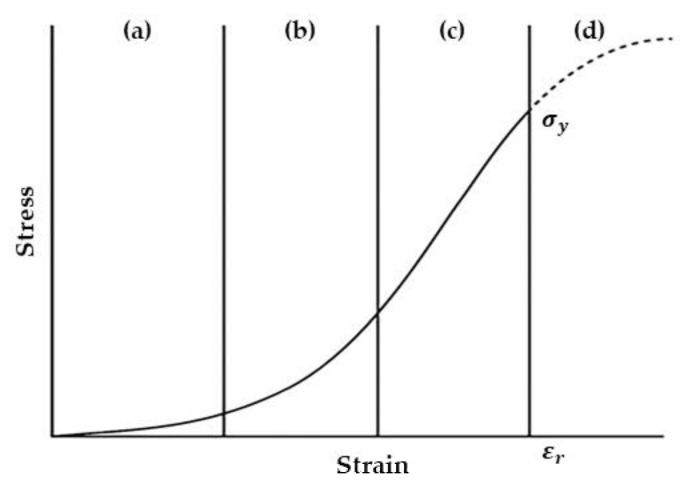
Typical stress-strain curve for soft tissue in the uniaxial tensile test: (**a**) elastin phase; (**b**) transition phase; (**c**) collagen phase; and (**d**) rupture [29]. ($\sigma_{y}\geq 2.0$ MPa, $\varepsilon_{r}\geq 2.0$ mm/mm).

Given the difficulty of procuring real human tissue samples, tissue-mimicking phantoms are of great importance for experimental biomedical research. Moreover, realistic tissue-mimicking phantoms are challenging to create. As in the previous introduction, 3D printing is advantageous for making these characteristic models. Therefore, our study aimed to mimic the mechanical properties of the aorta. The aorta serves as a regulator of blood pressure and flows throughout the cardiovascular system, through its mechanical properties and blood vessel functions. The aorta is generally classified into the adventitia, the media, and the intima and consists of smooth muscle cells, collagen fibers, and elastic lamina (Figure 1). Several studies have described the mechanical behavior of arteries as occurring in phases, as shown in Figure 2: (1) When subjected to low levels of deformation, the elastic lamina supports the load; (2) the collagen fibers divide the load as the deformation increases; and (3) in cases of high deformation, the collagen fibers support the load and break when their yield point is exceeded [27,28]. The tensile strength of normal arteries has been defined in previous studies as 2.0 to 3.0 MPa, and the strain at breaking was 2.0 to 2.5 mm/mm [27,28,29].

Crucial to successfully replicating the mechanical properties of arteries is the simulation of the elastic lamina and collagen fibers that distribute the load based on changes due to deformation. In arterial tissue, collagen fibers are irregularly twisted and oriented in patterns to address the increasing strain to support the load. In this study, we sought to imitate this behavior by applying a high-hardness material with a pattern, such as a lattice shape. In several studies, the stress-strain curves of the arteries varied in slope (i.e., their modulus of elasticity) depending on their intended function. Through our experiments and analyses, we determined the mechanical properties of several materials. We then applied the materials to the challenge of replicating the mechanical properties of the aortic wall by utilizing pattern design and 3D printing to overcome material limitations and provide a realistic facsimile.

## 2. Materials and Methods

### 2.1. Tensile Testing of the Base Materials

**Figure 3:**
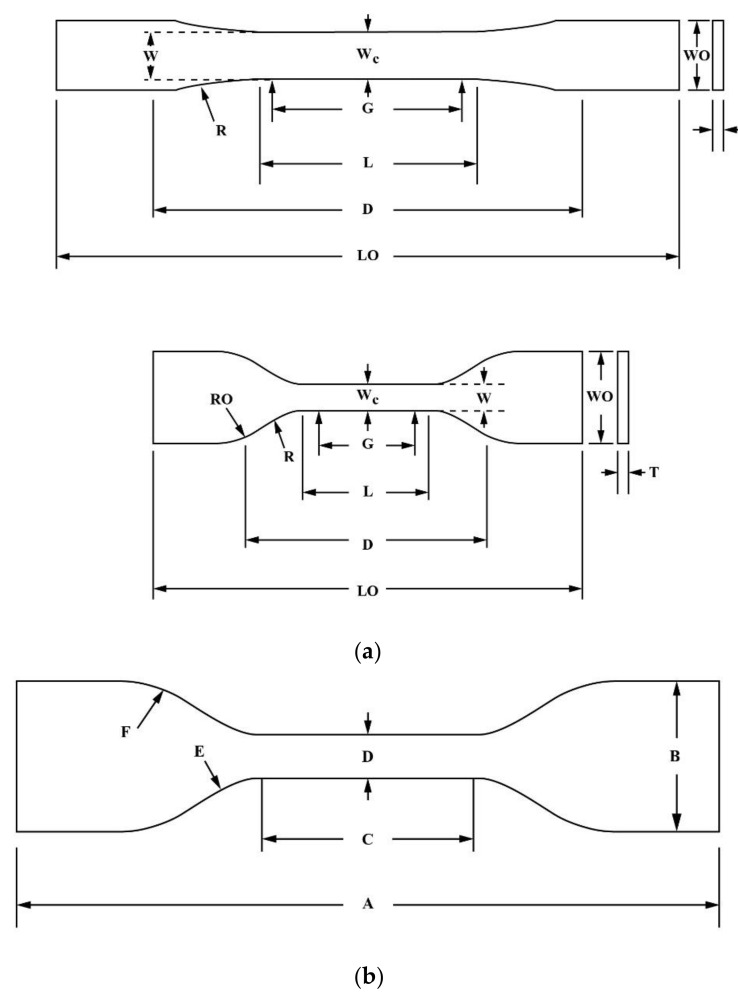
Specimen dimensions for (**a**) ASTM D638 and (**b**) ASTM D412 [36,37].

The materials used in the study were subjected to tensile testing to inform the design of suitable patterns and specimens. Agilus capable of printing to Objet500 connex3 (Stratasys, Ltd. Eden Prairie, MN, USA), and Dragonskin 30 silicone (Smooth-On, Inc., Macungie, PA, USA) were used as a base material. A detailed description of the material was in Table 1. VeroCyan capable of printing to Objet500 connex3, and TPU 95A, capable of printing to Ultimaker3 (Ultimaker B.V., Geldermalsen, The Netherlands) were used as pattern materials. The Agilus and VeroCyan could be printed simultaneously, the Dragonskin was used for printing a vascular phantom, and the TPU 95A was a flexible material capable of stable production using fused deposition modeling [30,31]. Three-millimeter-thick specimens of the Agilus and VeroCyan were tested per ASTM D638 (Standard Test Method for Tensile Properties of Plastics). ASTM D412 (Standard Test Methods for Vulcanized Rubber and Thermoplastic Elastomers—Tension) was followed for testing 2.5 mm thick specimens of the Dragonskin 30 and TPU 95A. The specimen dimensions are provided in Figure 3 and Table 2.

**Table 1:** Type and composition of materials, Food and Drug Administration approval [32,33,34,35].

Material	Type	Composition	FDA Approval
Agilus	Rubber-Like	Proprietary1	None
		Proprietary2	
		Proprietary3	
		Proprietary4	
		Glycerol, propoxylated, esters with acrylic acid	
		Acrylic acid, 2-hydroxyethyl ester	
		Stabilizer	
		2,6-Bis(1,1-Dimethylethyl)-4-Methyl-Phenol	
		Camphene	
		1,7,7-Trimethyltricyclo[2.2.1.02,6]heptane	
VeroCyan	Rigid	Proprietary1	None
		Proprietary2	
		Proprietary3	
		Proprietary4	
		Proprietary5	
		Proprietary6	
		Titanium dioxide	
		Camphene	
		Glycerol, propoxylated, esters with acrylic acid	
		Ethoxylated Trimethylolpropane Triacrylate	
		Acrylic acid	
Dragonskin 30	Silicon	No data	None
TPU 95A	Flexible	Thermoplastic polyurethane	None

The tensile tests were performed using an ST-1001 material testing machine (SALT CO, Ltd., Incheon, Korea), which applied tension to five specimens of each material at a rate of 50 mm/s, except VeroCyan. VeroCyan was tensioned at a rate of 5 mm/s according to the rigid plastic test method. The results are provided as stress-strain curves.

### 2.2. Patterns and Specimen Design

**Figure 4:**
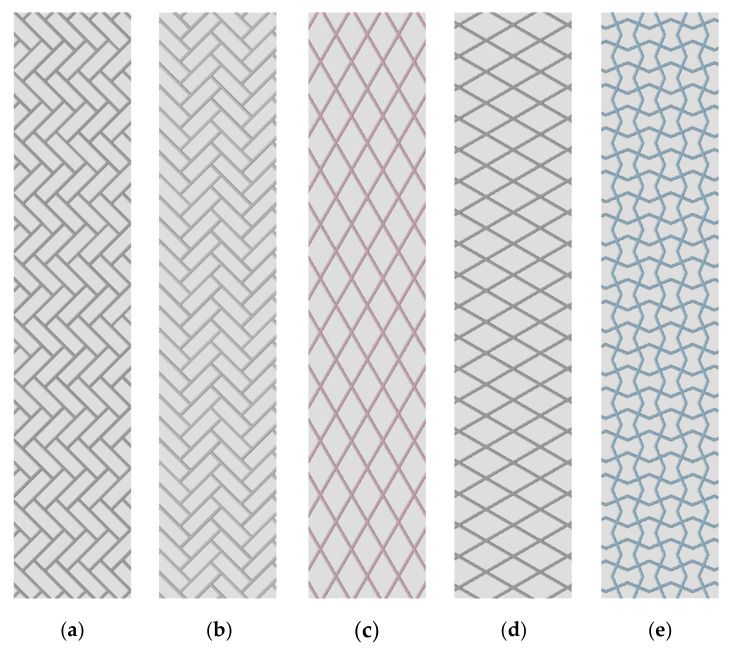
Pattern-embedded specimens: (**a**) Pattern A aligned with the major axis; (**b**) Pattern A aligned with the minor axis; (**c**) Pattern B aligned with the major axis; (**d**) Pattern B aligned with the minor axis; and (**e**) Pattern C.

One anisotropic 2D pattern (Pattern A) and two orthotropic patterns (Patterns B and C) were designed to have fiber diameters of 0.7 mm and 1.4 mm and a major axis length of 15 mm or less, based on the tensile test results. The minor axis length varied between the patterns, but all were less than 8 mm (Table 3). The specimens were produced by embedding the designed pattern inside a rectangular box. The anisotropic patterns were embedded in the specimen by matching the tensile direction, major axis, and minor axis. In contrast, the orthotropic patterns were embedded without regard for the major and minor axes (Figure 4). The Agilus–VeroCyan specimens were designed with a 150 mm × 30 mm box and a thickness of 3 mm, and the Dragonskin 30–TPU 95A specimens were designed with a 115 mm × 25 mm box and a thickness of 2.5 mm, based on the differences in tensile strength and strain of the base materials.

### 2.3. Tensile Testing of the Pattern-Embedded Specimens

**Figure 5:**
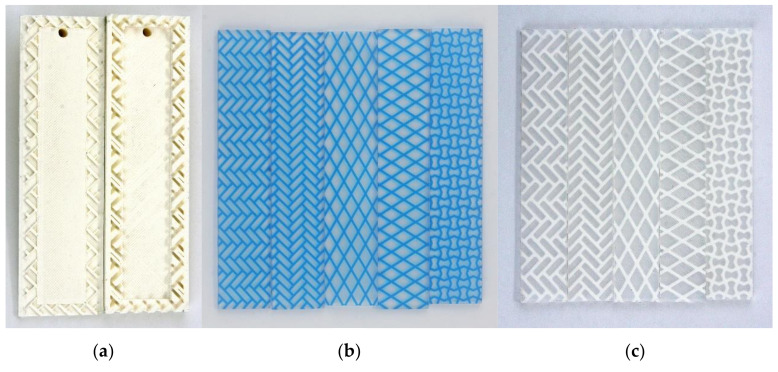
3D printed specimen mold and pattern-embedded specimens: (**a**) 3D printed specimen mold for Pattern A with major axis alignment; (**b**) 3D printed pattern-embedded specimens; and (**c**) silicon-molded pattern-embedded specimens.

The designed specimens were 3D printed, molded, and subjected to tensile tests. The Agilus (box) and VeroCyan (pattern) were printed simultaneously by the Objet500 connex3 (Stratasys, Ltd., Eden Prairie, MN, USA). The print direction was perpendicular to the tensile direction, and all specimens were produced in the same direction. Five Agilus–VeroCyan specimens were printed for each pattern. The Ultimaker3 printed the TPU 95A pattern for the Dragonskin 30–TPU 95A specimen. The mold was designed to center the pattern in the Dragonskin 30 silicone molding. The Ultimaker 3 printed all the pattern molds with polylactic acid, as shown in Figure 5. The two components comprising the Dragonskin 30 raw materials were placed in a syringe, and air bubbles were removed using the vacuum chamber. After placing the pattern on the mold and sealing it, it was filled with the Dragonskin 30 silicone using a two-component gun and cured for one day at room temperature. Five Dragonskin 30–TPU 95A specimens were also produced using the same procedure. The produced Agilus–VeroCyan and Dragonskin 30–TPU 95A specimens are shown in Figure 5.

**Figure 6:**
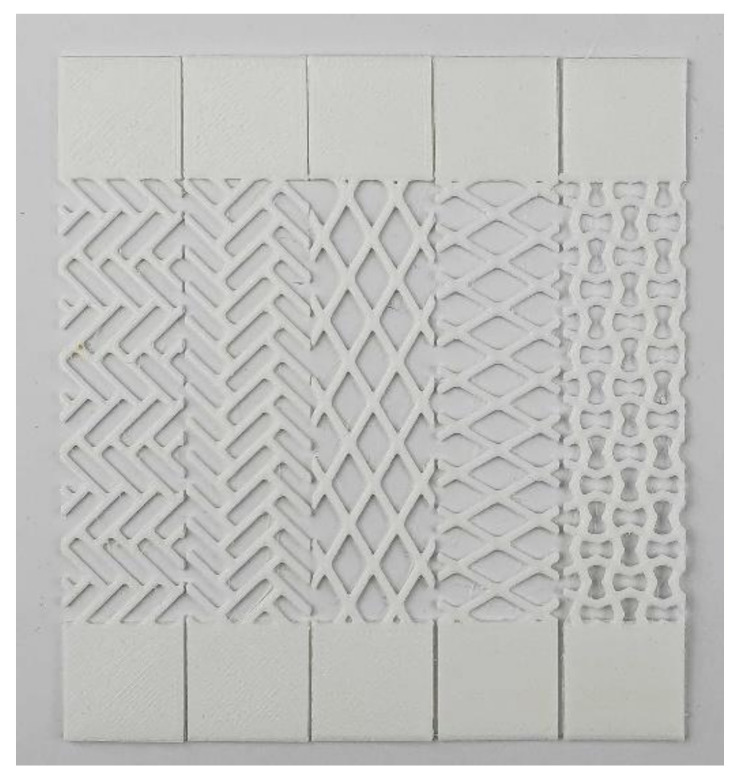
3D printed pattern-specimens of TPU 95A.

For examining the effect of patterning, the pattern was printed on the TPU 95A, and a tensile test was performed. Five TPU 95A-patterned specimens were printed for each of the five patterns evaluated in this study and with two thicknesses (0.7 mm and 1.4 mm) using the Ultimaker3 (Figure 6). The tensile testing was performed using the ST-1001 testing machine, which pulled each specimen at 50 mm/s, yielding a stress-strain curve for each specimen type.

### 2.4. Evaluation

In this study, the tensile strength of the base materials (Agilus, VeroCyan, Dragonskin 30, and TPU 95A) and the tensile test results of the pattern-embedded specimens were analyzed. Because the time axis for each test type was not the same between the tensile test result data, the average and standard deviation values were obtained through polynomial fitting. The average and standard deviations of the R-squared and root-mean-square error (RMSE) values of the fourth-order polynomials derived for each material tested are shown in Tables S1–S3.

The averages of the tensile strength and fracture strain values for the five test results for each pattern-embedded specimen was compared with the known aortic tensile strength and fracture strain values (2.0–3.0 MPa and 2.0–2.3 mm/mm, respectively). The elastic modulus was obtained from the slope of the fitted polynomial based on reference. MATLAB R2015a (The MathWorks, Inc., Torrance, CA, USA) was used to analyze the result and produce the graphs.

## 3. Results

**Figure 7:**
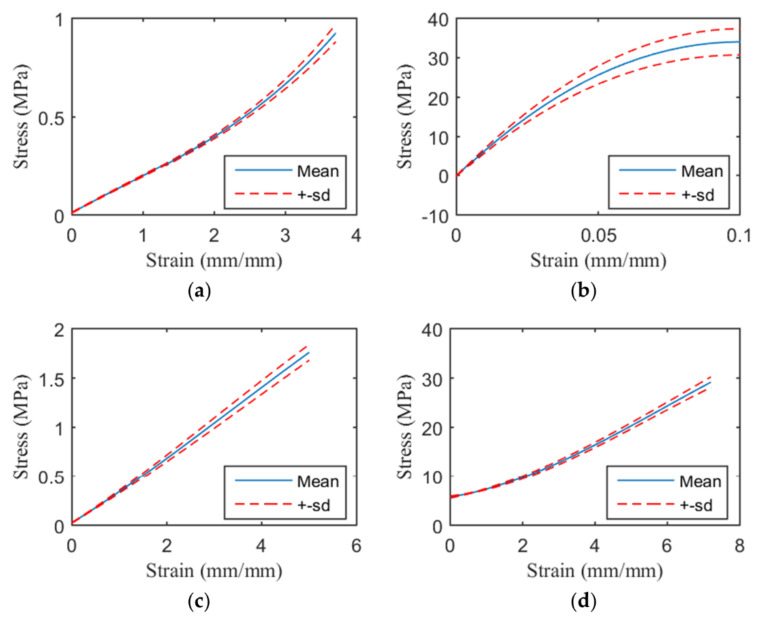
Stress-strain curves of the base materials. Mean stress-strain curves before yield for the: (**a**) Agilus; (**b**) VeroCyan; (**c**) Dragonskin 30; and (**d**) TPU 95A.

The average tensile strength and standard deviation of the Agilus, VeroCyan, Dragonskin 30, and TPU 95A were 1.00 ± 0.05 MPa, 34.08 ± 3.31 MPa, 2.03 ± 0.17 MPa, and 36.71 ± 3.85 MPa, respectively; the average strains and standard deviations were 3.96 ± 0.12 mm/mm, 0.38 ± 0.08 mm/mm, 5.82 ± 0.46 mm/mm, and 9.55 ± 1.25 mm/mm, respectively. The stress-strain curves for these four materials are shown in Figure 7.

**Figure 8:**
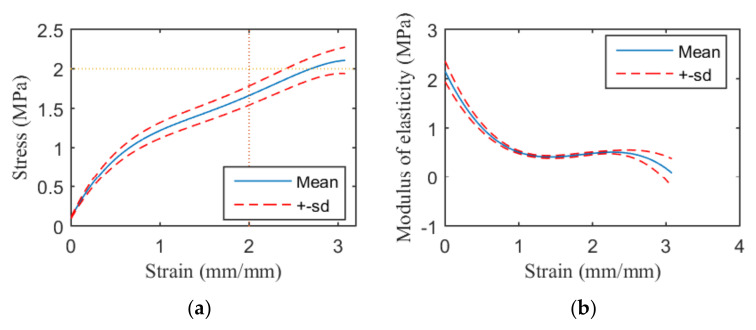
Stress-strain curve and modulus of elasticity of the Pattern C Dragonskin 30–TPU 95A specimens with a 1.4 mm pattern diameter: (**a**) stress-strain curve before yield and (**b**) modulus of elasticity.

The Dragonskin 30–TPU 95A Pattern C specimens with the 1.4 mm pattern diameter met or exceeded the mechanical properties of a human aorta, as shown in Figure 8. The stress-strain curves of Agilus–VeroCyan and Dragonskin 30–TPU 95A specimens with pattern diameters of 0.7 mm are shown in Figures S1 and S2, respectively. The stress-strain curves of the Agilus–VeroCyan and Dragonskin 30–TPU 95A specimens with 1.4 mm pattern diameters are shown in Figures S3 and S4, respectively. The stress-strain curves of the Pattern C TPU 95A specimens are shown in Figures S5 and S6. The average tensile strength and strain at breaking for all the specimens and patterns are provided in Table 4. The elastic modulus graphs for each tensile test are shown in Figures S7–S12.

## 4. Discussion

The obtained values of the tensile strength, strain at break, and modulus of elasticity of the Agilus–VeroCyan and Dragonskin 30–TPU 95A specimens with embedded patterns were compared to the mechanical properties of the aortic wall. The Dragonskin 30–TPU 95A specimen met the criteria set for strain at breaking but had insufficient tensile strength. The Agilus–VeroCyan specimens demonstrated lower strain at break than the Dragonskin 30–TPU 95A specimens for all the patterns. Increasing the diameter of the pattern increased the tensile strength and the stiffness but reduced the strain at break. The mechanical properties of the specimens differed from those of the aorta. However, rupturing is considered a critical issue for the real-world application of these materials as human tissues, so the increased tensile strength was considered sufficient.

The modulus of elasticity between the two inflection points increased from the 0.7 mm thick Agilus–VeroCyan A-major specimen to the same B-minor specimen, followed by the 1.4 mm thick Dragonskin 30–TPU 95A B-minor specimen. Although none of these patterns met the tensile strength criterion, the 1.4 mm Dragonskin 30–TPU 95A with the B-minor pattern met the strain at break criterion; however, the elastic modulus change was not significant. There were no inflection points for some of the patterns, which could indicate that the differences in the elastic moduli between the base materials were minimal or that one of the materials dominated the performance. The stress-strain curve and elastic modulus graph of the TPU 95A confirmed that the mechanical properties of the material did not vary significantly between the patterns. Although differences existed between the tensile strength and strain values obtained for each pattern, there were no significant variations in the shapes of the graphs. Although the mechanical property criteria were satisfied by the Pattern C samples, because the aorta has a strain rate of approximately 3% to 8% during systole, adequate reproduction of aortic behavior requires that the elastic modulus properties also be satisfied [38,39].

By design, the pattern with the most extensive deformation until the fibers of the pattern were aligned close to a straight line is the B-minor, but in all results, the B-minor strain was not the largest. In the Agilus–VeroCyan specimen, VeroCyan was rigid, so the B-minor strain was the highest according to the pattern design. Moreover, because Agilus and VeroCyan were well bonded, the Agilus tended to rupture at the same time as VeroCyan ruptures. On the other hand, in the Dragonskin 30–TPU 95A specimen, both materials are very flexible and do not bond well during the molding process. For this reason, it showed strain at break, which was not greatly restricted by the pattern. Besides, in the base material tensile test, Dragonskin 30 showed a higher strain than TPU 95A, but in the Dragonskin 30–TPU 95A specimen, the Dragonskin tended to rupture first. That is presumed to be because the two materials were not bonded, and cracks (bubbles) occurred during molding. When testing with other materials or fabricate phantoms, these results should be considered.

Although the inherent differences in mechanical behaviors between soft tissues and the polymers, such as VeroBlackPlus, could be narrowed, the polymer did not demonstrate a wide range of applicability, unlike human tissues under complex loading [18,19]. In this study, we sought to overcome this limitation by combining materials and applying two-dimensional patterns. Through this effort, we confirmed the feasibility of producing differences in the circumferential and longitudinal tensile strengths, as occurs in aortic tissue, through the application of 3D printing and pattern embedment. Coulter et al. made aortic valves by extruding silicone on a five-axis printer [40]. They revealed that the shear strength and fatigue strength could be increased by applying a pattern to the aortic valve. Recently, Kampen et al. applied 3D printing to the axis of rotation and fabricated hollow tubular scaffolds with full control over design and geometry [41]. They concluded that mechanical properties such as flexibility and compression modulus could be controlled depending on the grid design. Our research was to create a flat pattern and insert it into the specimen. Applying Kampen’s research to ours is expected to be able to control more diverse characteristics.

Uniaxial tensile testing has demonstrated that the mechanical properties of TangoPlus, a flexible 3D printer material, are superior to those of polydimethylsiloxane, which is commonly used for vascular phantoms [14]. Another study printed a descending aorta of TangoPlus and confirmed its ability to swell by changing its internal pressure, which indicated its suitability for applying an aneurysm phantom model [13]. The Agilus and Dragonskin 30 evaluated in the present study demonstrated higher tensile strength and strain at breaking than TangoPlus (Figure 7). The TPU 90A had the highest tensile strength and strain at breaking among the examined materials but is too hard and stiff to be the sole material used for an artificial aorta. The patterned TPU 95A met the tensile strength criteria based on the mechanical properties of the aorta; however, for this material to be used as a phantom, a membrane would be required to contain the fluid. Gore-Tex, which is widely used for artificial vascular grafts, has different mechanical properties than does the aorta, with a tensile strength of 14.03 ± 0.72 MPa at a strain of 27.8% and an elastic modulus of 31.61 ± 4.76 MPa [42,43]. Although it is not likely to rupture in the human body and is used because of its high biocompatibility, Gore-Tex is not suitable for aortic phantoms. However, assuming that it was inserted into the human body, there were no materials like Gore-Tex. The materials used in this study were not medical grade. Instead, it could be used as an implant by coating it with medical-grade silicone or inserting a designed pattern inside the silicone to create an artificial aorta.

Despite the positive results obtained, there were some limitations to this study. Firstly, the embedded patterns prevented tensile testing from being performed precisely according to the specifications. The processes followed the ASTM D638 and D412 standards, except for constructing the specimen with the embedded patterns. Therefore, the design and verification of the test method for specimens with embedded patterns should be undertaken to obtain results that represent their material properties more accurately. Furthermore, the tensile test results obtained for plate-shaped specimens could only approximate the mechanical properties of the cylindrical aorta. If tubular printing is used to create a pattern-embedded aorta, more accurate results will be obtained [41]. However, it also is needed to apply a suitable verification method.

Although the tensile strength and strain at break criteria of the aorta could be met, there were differences in the tensile strength and modulus of elasticity characteristics depending on the strain. Because only one of the tested patterns satisfied the criteria, a different combination of materials or materials with different properties will need to be investigated. Further research is required to develop new patterns that could match the mechanical behavior of tissues. Finally, a flow phantom or artificial aorta would be subjected to repeated loads, so studying the fatigue limits and characteristics is also required.

## 5. Conclusions

We proposed a new method to imitate the mechanical characteristics of the aortic wall by using two materials having different physical properties and embedded patterns instead of using a single 3D printed material. We successfully replicated the tensile strength and strain of the aorta and identified the possibility of producing a more realistic phantom. Further, the results could be applied to the mimicry of other human tissues.

## Figures and Tables

**Table 2:** Specimen dimensions (units: mm).

Dimension	Agilus ASTM D638 Type I	VeroCyan ASTM D638 Type IV	Dragonskin 30 and TPU 95A ASTM D412 Type I
Width of narrow section	13	6	6
Length of narrow section	57	33	33
Minimum overall width	19	19	25
Minimum overall length	165	115	115
Gage length	50	25	25
Distance between grips	115	65	-
Radius of fillet	76	14	14
Outer radius	-	25	25

**Table 3:** Pattern designs.

Name	Type	Fiber Diameters (mm)	Major Axis (mm)	Minor Axis (mm)
Pattern A	Anisotropic	0.7 and 1.4	15.66	5.66
Pattern B	Orthotropic		13.86	8.00
Pattern C	Orthotropic		11.09	11.09

**Table 4:** Mean yield stress and strain at break (± standard deviation) for each pattern design.

Material	Diameter (mm)	Pattern Type	Mean Yield Stres (MPa)	Strain at Break (mm/mm)
Agilus-VeroCyan	0.7	A major	0.64 ± 0.03	0.83 ± 0.52
		A minor	0.50 ± 0.01	1.29 ± 0.04
		B major	0.79 ± 0.05	0.60 ± 0.22
		B minor	0.59 ± 0.01	1.50 ± 0.05
		C	0.63 ± 0.04	0.76 ± 0.16
Dragonskin 30–TPU 95A		A major	1.40 ± 0.09	4.20 ± 0.45
		A minor	1.27 ± 0.10	3.78 ± 0.26
		B major	1.45 ± 0.11	3.85 ± 0.38
		B minor	1.29 ± 0.08	3.04 ± 0.30
		C	1.03 ± 0.06	3.17 ± 0.31
Agilus-VeroCyan	1.4	A major	1.19 ± 0.07	0.50 ± 0.10
		A minor	1.09 ± 0.07	0.39 ± 0.08
		B major	2.88 ± 0.18	0.32 ± 0.06
		B minor	0.62 ± 0.02	0.74 ± 0.05
		C	2.15 ± 0.12	0.27 ± 0.03
Dragonskin 30–TPU 95A		A major	1.54 ± 0.12	2.70 ± 0.34
		A minor	1.40 ± 0.06	2.90 ± 0.23
		B major	1.85 ± 0.08	2.58 ± 0.12
		B minor	1.09 ± 0.10	2.49 ± 0.22
		C	2.15 ± 0.15	3.18 ± 0.05

## Supplementary Materials

The following are available online at https://www.mdpi.com/1996-1944/13/21/5042/s1. Table S1: Mean and standard deviation of the R-square and RMSE values of the polynomial fitting of the tensile test results of the base materials, Table S2: Mean and standard deviation of the R-square and RMSE values of the polynomial fitting of the tensile test results of the specimens with embedded patterns, Table S3: Mean and standard deviation of the R-square and RMSE values from the polynomial fitting of the tensile test results of the patterned TPU 95A specimens, Figure S1: Mean stress-strain curves of the Agilus–VeroCyan specimens with the 0.7 mm embedded patterns, Figure S2: Mean stress-strain curves of the Dragonskin 30–TPU 95A specimens with the 0.7 mm embedded patterns, Figure S3: Mean stress-strain curves of the Agilus–VeroCyan specimens with the 1.4 mm embedded patterns, Figure S4: Mean stress-strain curves of the Dragonskin 30–TPU 95A specimens with the 1.4 mm embedded patterns, Figure S5: Mean stress-strain curves of the TPU 95A specimens with the 0.7 mm embedded patterns, Figure S6: Mean stress-strain curves of the TPU 95A specimens with the 1.4 mm patterns, Figure S7: Modulus of elasticity of the Agilus–VeroCyan specimens with the 0.7 mm embedded patterns, Figure S8: Modulus of elasticity of the Dragonskin 30–TPU 95A specimens with the 0.7 mm embedded patterns, Figure S9: Modulus of elasticity of the Agilus–VeroCyan specimens with the 1.4 mm embedded patterns, Figure S10: Modulus of elasticity of the Dragonskin 30–TPU 95A specimens with the 1.4 mm embedded patterns, Figure S11: Modulus of elasticity of the TPU 95A specimens with the 0.7 mm embedded patterns, Figure S12: Modulus of elasticity of the TPU 95A specimens with the 1.4 mm patterns.
