# Supplementary material for: Mimicking the Mechanical Properties of Aortic Tissue with Pattern-Embedded 3D Printing for a Realistic Phantom
Source: Materials (Basel). 2020 Nov 9;13(21):5042. doi: 10.3390/ma13215042 (PMC7664885; doi:10.3390/ma13215042)
Supplement: Supplementary file 1 [file materials-13-05042-s001.pdf]

materials

MDPI

Article

Mimicking the Mechanical Properties of Aortic Tissue with Pattern-Embedded 3D Printing for a Realistic Phantom

Jaeyoung Kwon [1], Junhyeok Ock [1] and Namkug Kim [2,*]

[1] Department of Biomedical Engineering, Asan Medical Center, University of Ulsan College of Medicine, 388-1 Pungnap2-dong, Songpa-gu, 138-828 Seoul, Korea; nisroeld2@gmail.com

[2] Department of Convergence Medicine, Asan Medical Institute of Convergence Science and Technology, Asan Medical Center, University of Ulsan College of Medicine, 388-1 Pungnap2-dong, Songpa-gu, 138-828 Seoul, Korea; jhock8755@gmail.com

* Correspondence: nkkim@amc.seoul.kr; Tel.: +82-2-3010-6573

Table 1. Mean and standard deviation of the R-square and RMSE values of the polynomial fitting of the tensile test results of the base materials.

Materials	R-Square	RMSE
Agilus	0.9999 ± 0	0.0127 ± 0.0008
VeroCyan	0.9957 ± 0.0008	0.0019 ± 0.0002
Dragonskin 30	0.9999 ± 0.0001	0.0044 ± 0.0031
TPU 94A	0.9962 ± 0.0007	0.5998 ± 0.1136

Table 2. Mean and standard deviation of the R-square and RMSE values of the polynomial fitting of the tensile test results of the specimens with embedded patterns.

Materials	Diameter (mm)	Pattern Type	R-Square	RMSE
Agilus-VeroCyan	0.7	A major	0.9996 ± 0.0004	0.0034 ± 0.0021
		A minor	0.9947 ± 0.0034	0.0094 ± 0.0030
		B major	0.9984 ± 0.0021	0.0075 ± 0.0047
		B minor	0.9988 ± 0.0005	0.0064 ± 0.0012
		C	0.9998 ± 0.0001	0.0023 ± 0.0006
Dragonskin 30–TPU 94A		A major	0.9997 ± 0.0004	0.0049 ± 0.0013
		A minor	0.9999 ± 0	0.0035 ± 0.0004
		B major	0.9995 ± 0.0004	0.0082 ± 0.0029
		B minor	0.9996 ± 0.0004	0.0062 ± 0.0028
		C	0.9995 ± 0.0002	0.0075 ± 0.0020
Agilus-VeroCyan	1.4	A major	0.9999 ± 0.0001	0.0034 ± 0.0013
		A minor	0.9998 ± 0.0001	0.0043 ± 0.0003
		B major	0.9998 ± 0.0002	0.0101 ± 0.0059
		B minor	0.9993 ± 0.0011	0.0034 ± 0.0035
		C	0.9998 ± 0.0002	0.0094 ± 0.0023
Dragonskin 30–TPU 94A		A major	0.9995 ± 0.0002	0.0105 ± 0.0030
		A minor	0.9998 ± 0.0002	0.0049 ± 0.0022
		B major	0.9998 ± 0.0001	0.0060 ± 0.0014
		B minor	0.9990 ± 0.0007	0.0101 ± 0.0045
		C	0.9986 ± 0.0010	0.0178 ± 0.0059

Table 3. Mean and standard deviation of the R-square and RMSE values from the polynomial fitting of the tensile test results of the patterned TPU 94A specimens.

Diameter (mm)	Pattern Type	R-Square	RMSE
0.7	A major	0.9983 ± 0.0004	0.0476 ± 0.0001
	A minor	0.9994 ± 0.0001	0.0216 ± 0.0048
	B major	0.9974 ± 0.0004	0.0661 ± 0.0018
	B minor	0.9992 ± 0.0001	0.0281 ± 0.0010
	C	0.9986 ± 0.0002	0.0613 ± 0.0032
1.4	A major	0.9987 ± 0.0001	0.0373 ± 0.0011
	A minor	0.9999 ± 0	0.0084 ± 0.0005
	B major	0.9983 ± 0.0001	0.0631 ± 0.0036
	B minor	0.9969 ± 0.0004	0.0556 ± 0.0048
	C	0.9978 ± 0.0003	0.0799 ± 0.0051

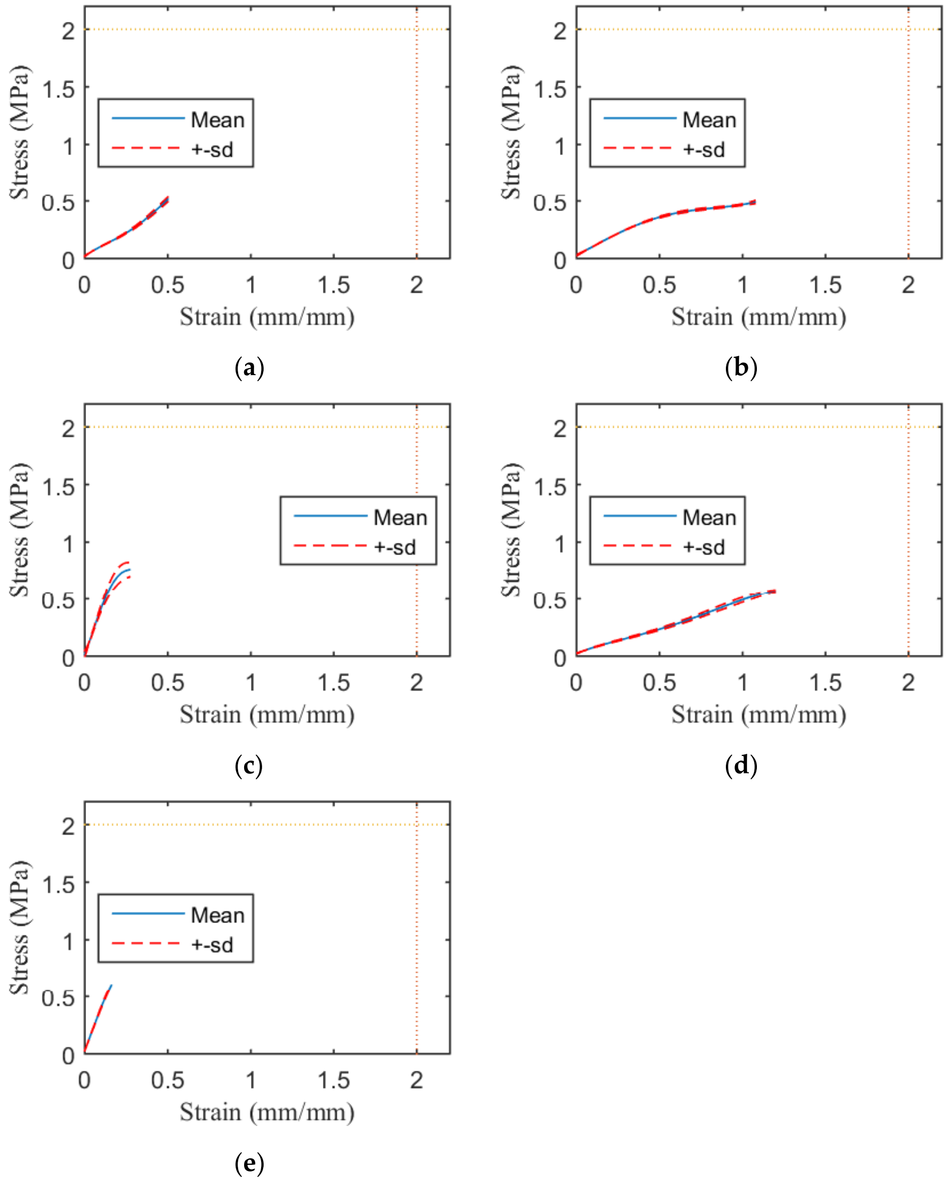

(**a**) (**b**) (**c**) (**d**) (**e**)

Figure 1. Mean stress-strain curves of the Agilus–VeroCyan specimens with the 0.7 mm embedded patterns: (**a**) Pattern A-major; (**b**) Pattern A-minor; (**c**) Pattern B-major; (**d**) Pattern B-minor; (**e**) Pattern C before yield.

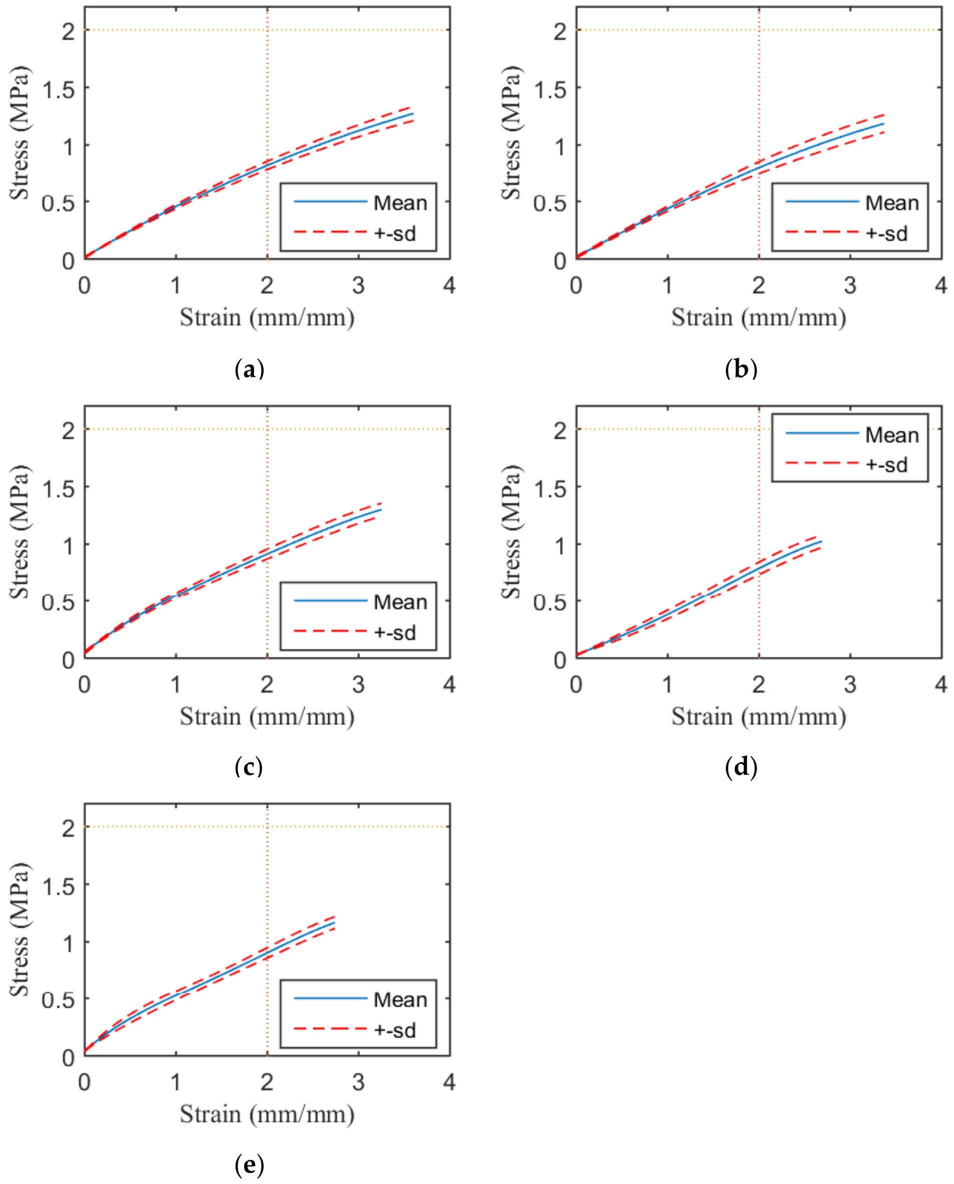

Figure 2. Mean stress-strain curves of the Dragonskin 30–TPU 94A specimens with the 0.7 mm embedded patterns: (**a**) Pattern A-major; (**b**) Pattern A-minor; (**c**) Pattern B-major; (**d**) Pattern B-minor; (**e**) Pattern C before yield.

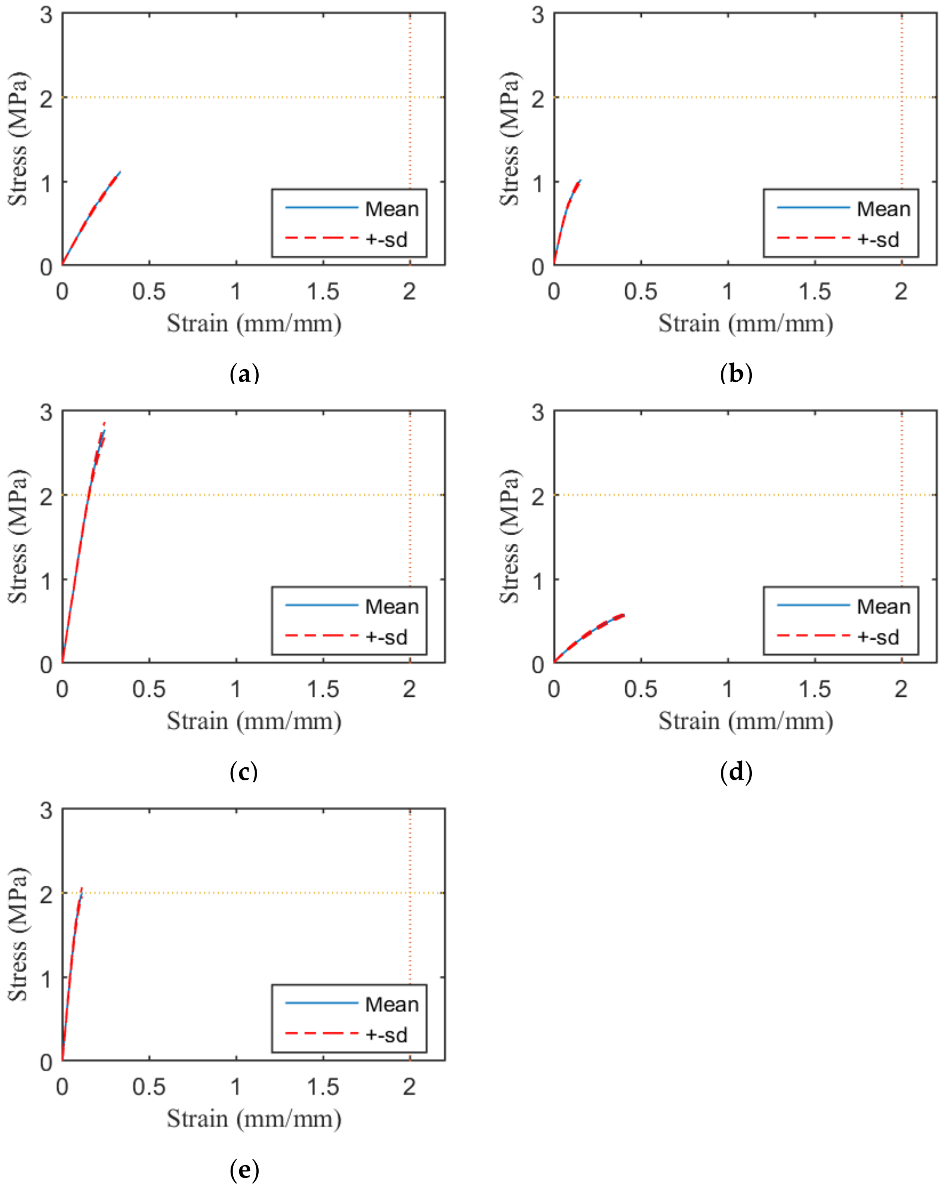

Figure 3. Mean stress-strain curves of the Agilus–VeroCyan specimens with the 1.4 mm embedded patterns: (**a**) Pattern A-major; (**b**) Pattern A-minor; (**c**) Pattern B-major; (**d**) Pattern B-minor; (**e**) Pattern C before yield.

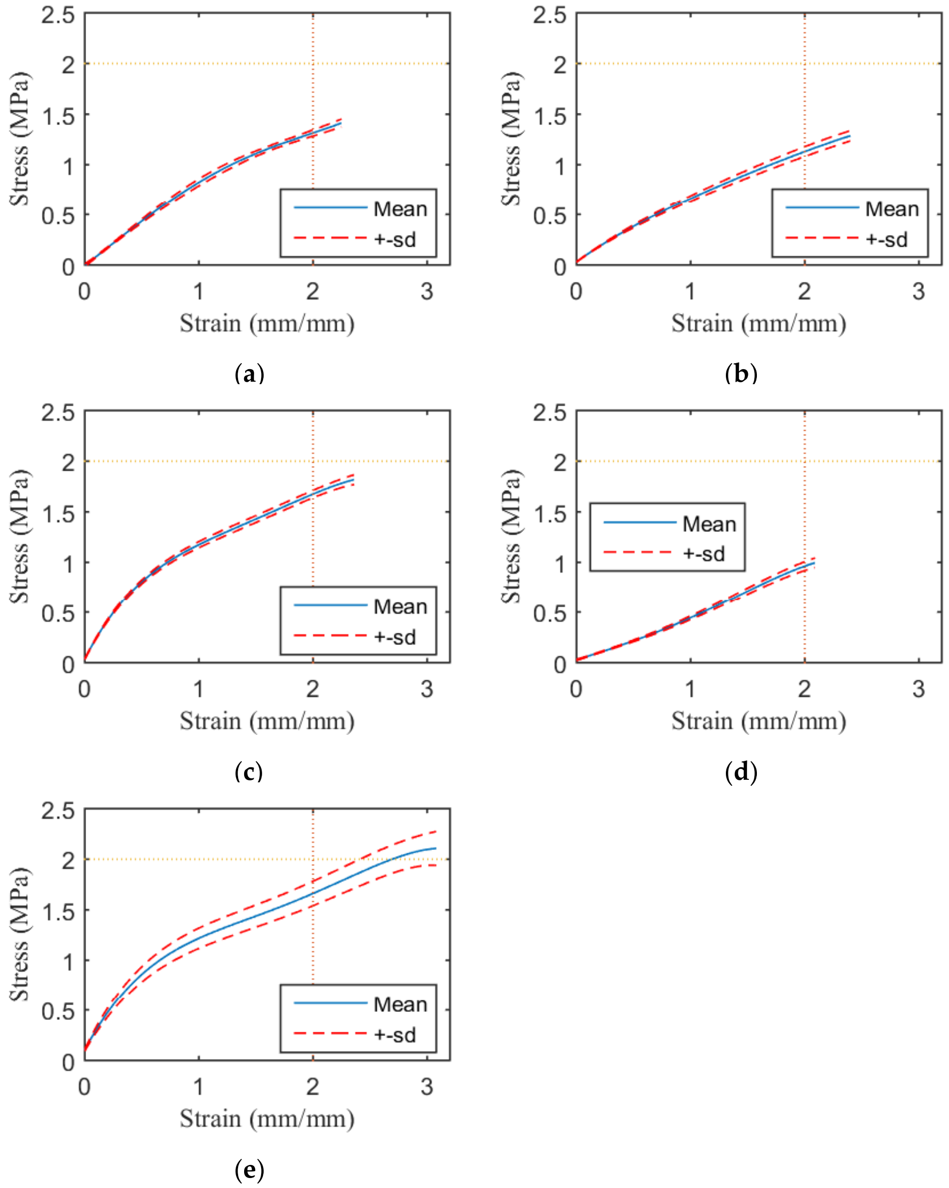

Figure 4. Mean stress-strain curves of the Dragonskin 30–TPU 94A specimens with the 1.4 mm embedded patterns: (**a**) Pattern A-major; (**b**) Pattern A-minor; (**c**) Pattern B-major; (**d**) Pattern B-minor; (**e**) Pattern C before yield.

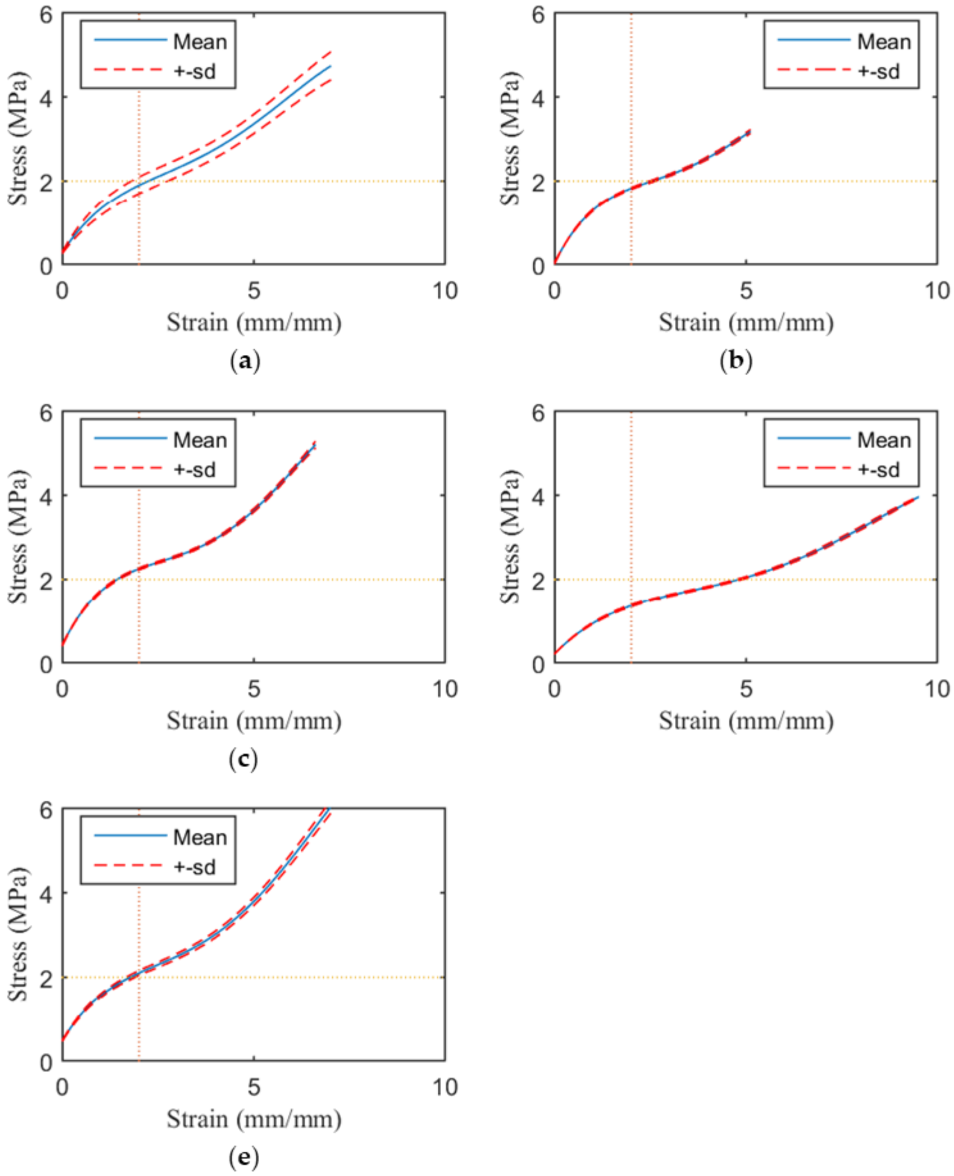

Figure 5. Mean stress-strain curves of the TPU 94A specimens with the 0.7 mm embedded patterns: (**a**) Pattern A-major; (**b**) Pattern A-minor; (**c**) Pattern B-major; (**d**) Pattern B-minor; (**e**) Pattern C before yield.

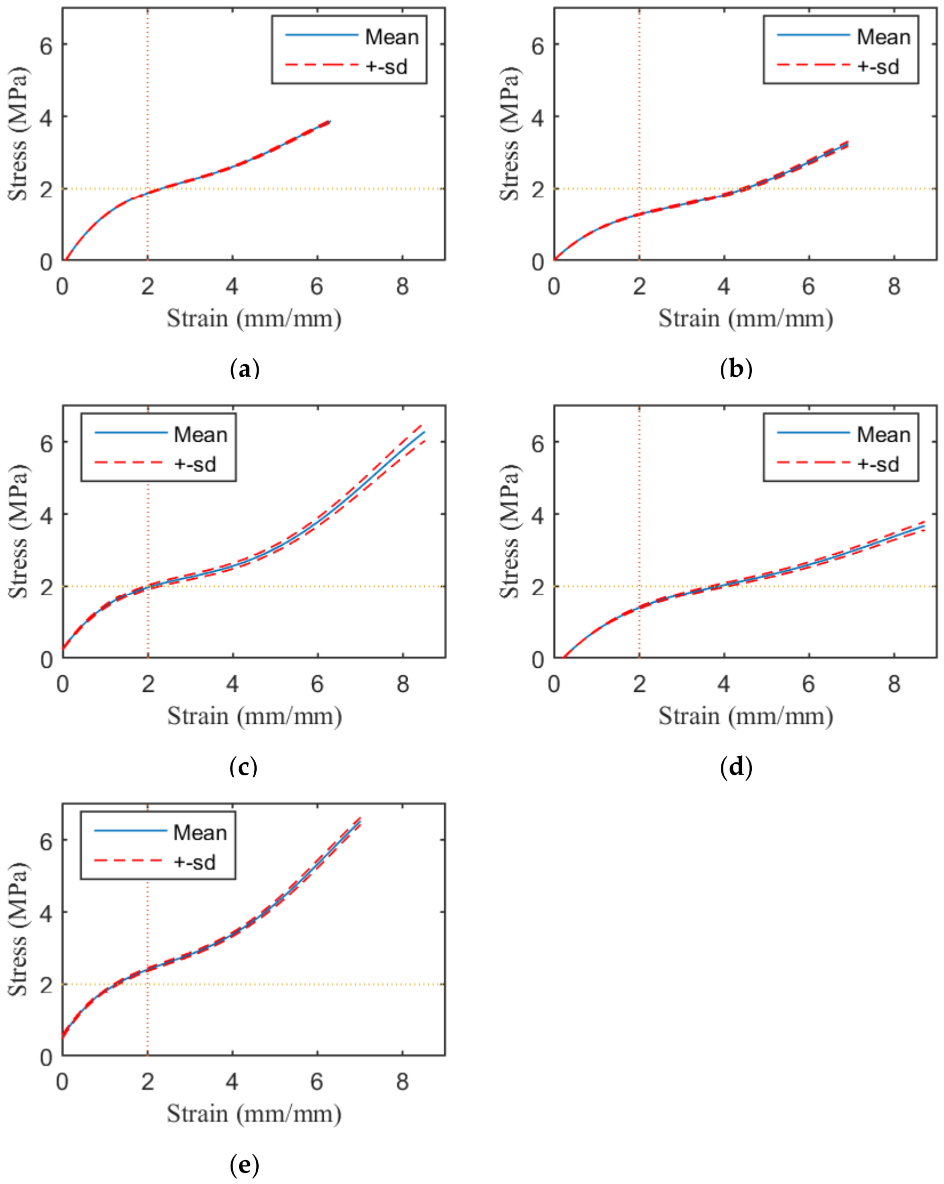

Figure 6. Mean stress-strain curves of the TPU 94A specimens with the 1.4 mm patterns: (**a**) Pattern A-major; (**b**) Pattern A-minor; (**c**) Pattern B-major; (**d**) Pattern B-minor; (**e**) Pattern C before yield.

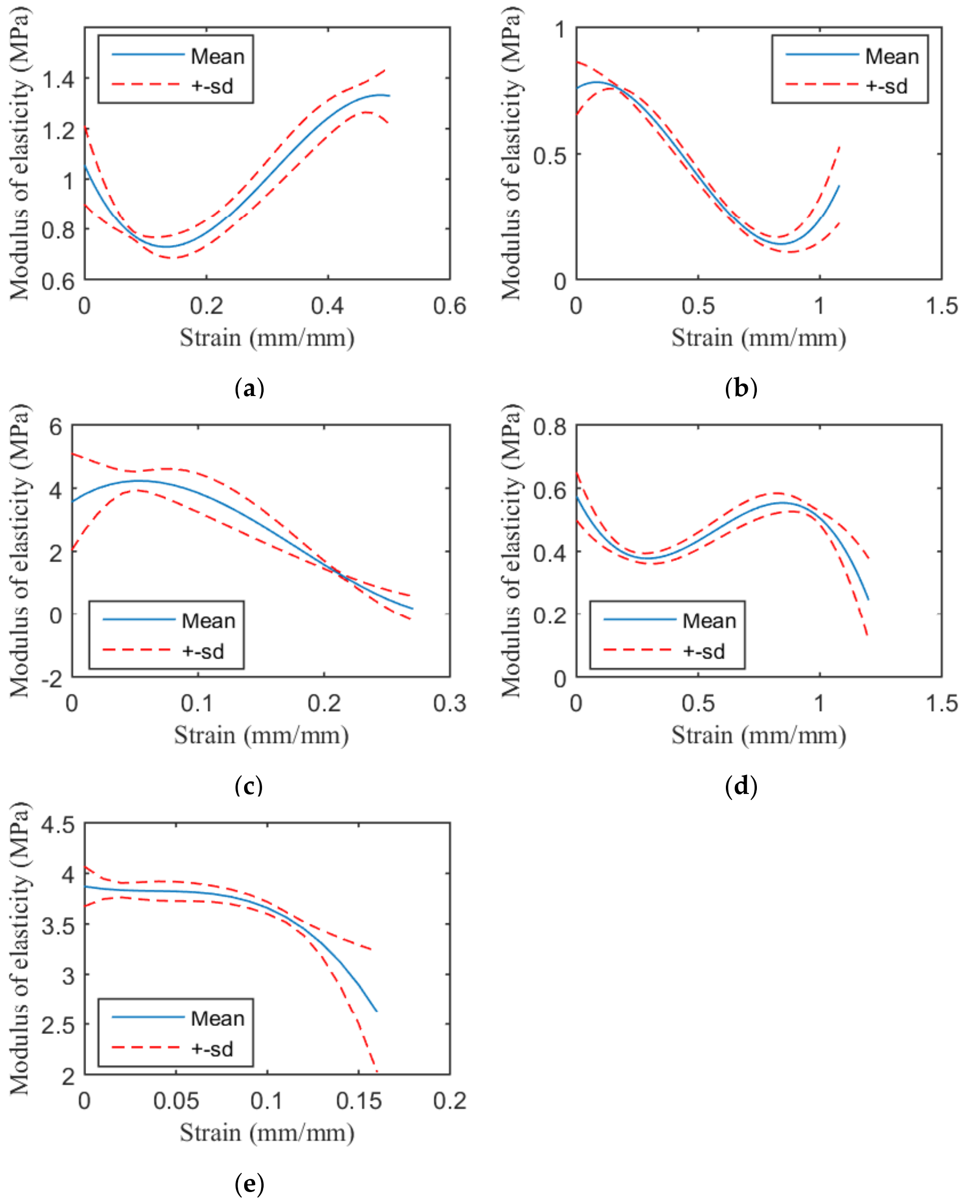

Figure S7. Modulus of elasticity of the Agilus–VeroCyan specimens with the 0.7 mm embedded patterns: (**a**) Pattern A-major; (**b**) Pattern A-minor; (**c**) Pattern B-major; (**d**) Pattern B-minor; (**e**) Pattern C.

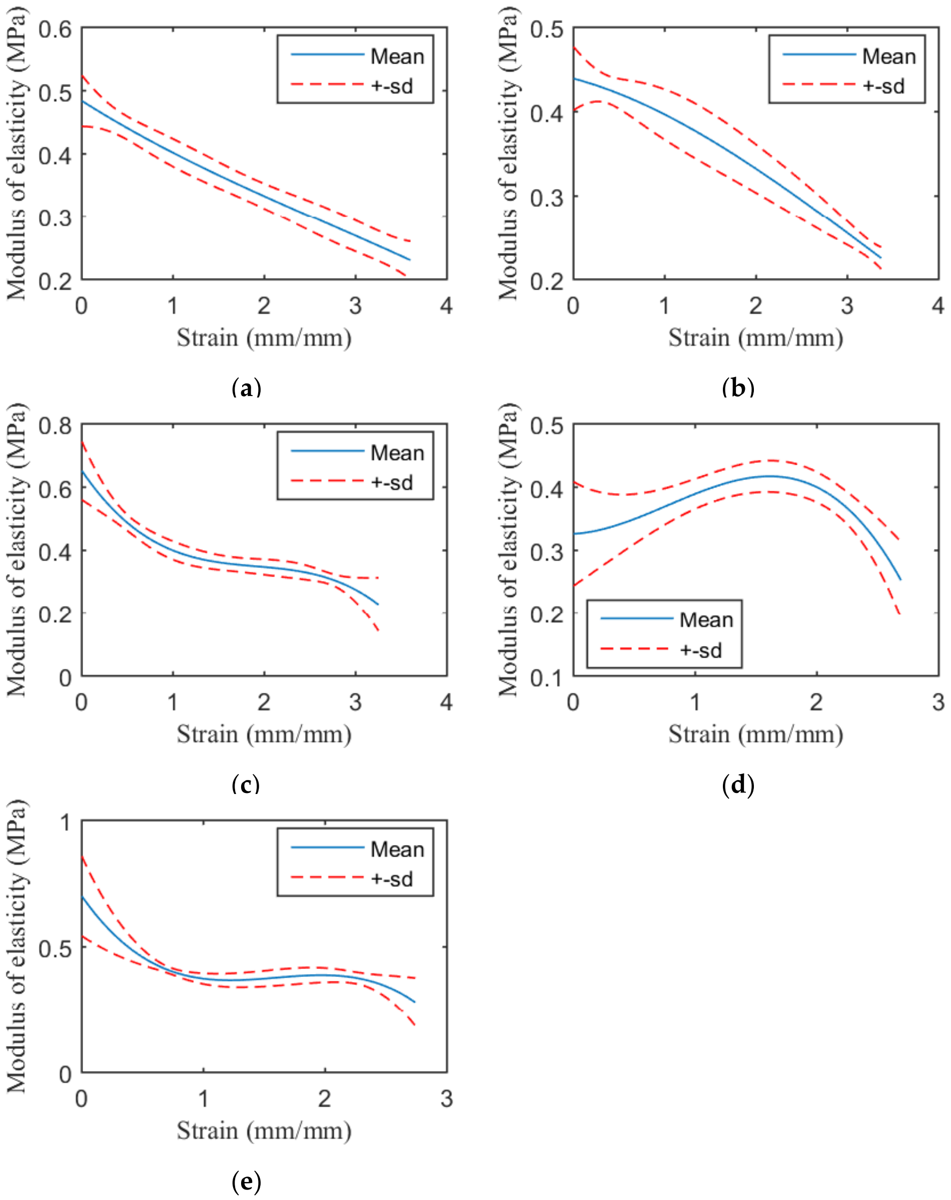

Figure 8. Modulus of elasticity of the Dragonskin 30–TPU 94A specimens with the 0.7 mm embedded patterns: (**a**) Pattern A-major; (**b**) Pattern A-minor; (**c**) Pattern B-major; (**d**) Pattern B-minor; (**e**) Pattern C.

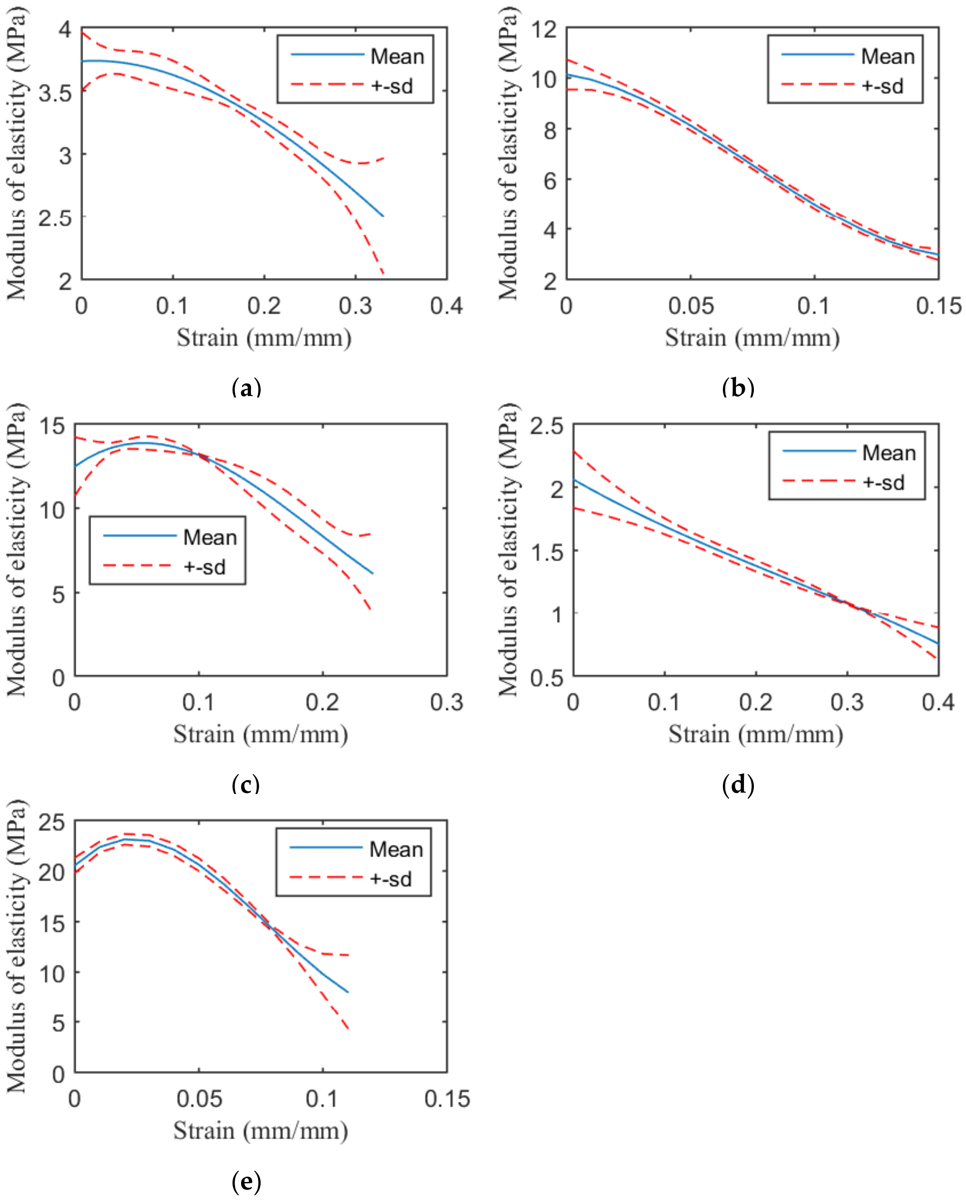

Figure 9. Modulus of elasticity of the Agilus–VeroCyan specimens with the 1.4 mm embedded patterns: (**a**) Pattern A-major; (**b**) Pattern A-minor; (**c**) Pattern B-major; (**d**) Pattern B-minor; (**e**) Pattern C.

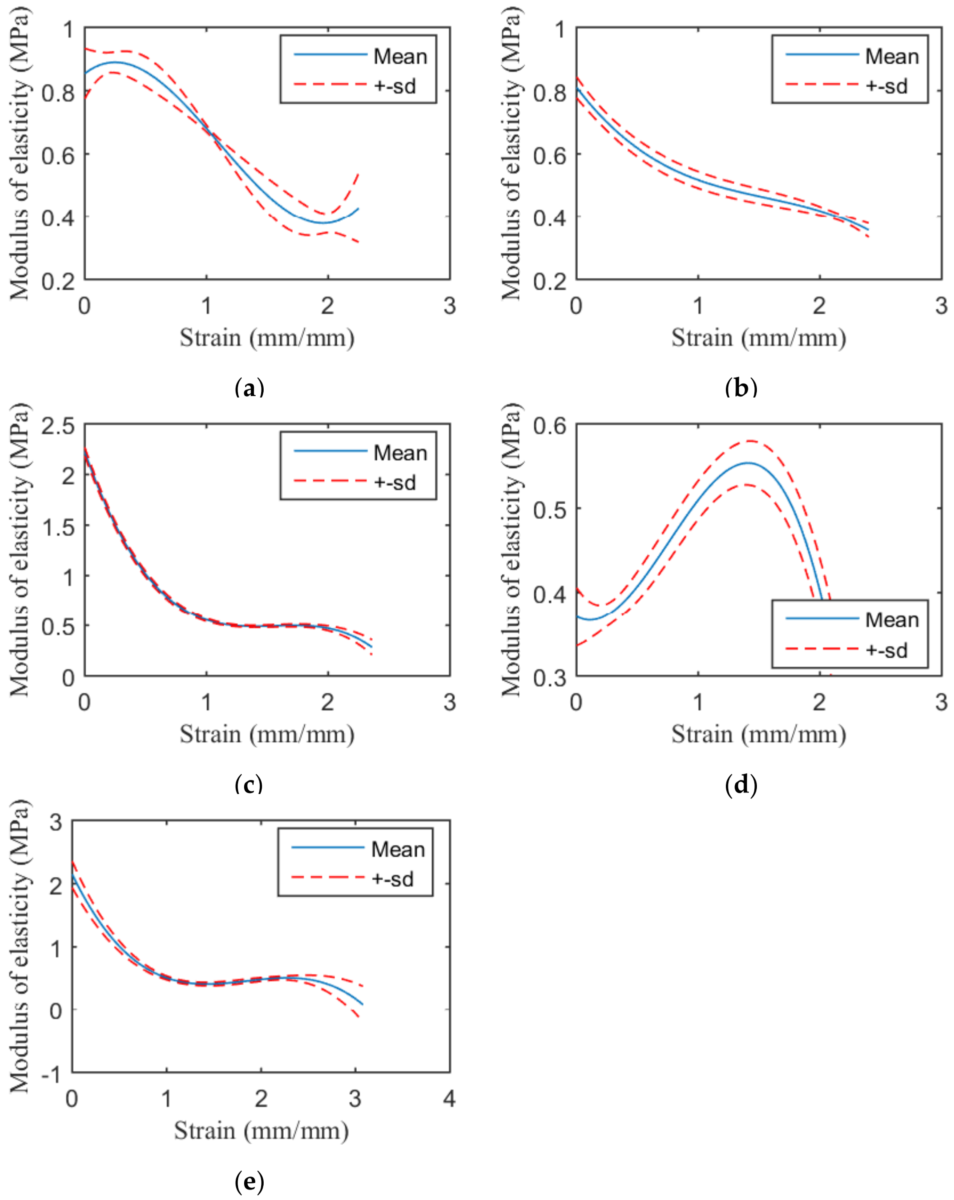

Figure 10. Modulus of elasticity of the Dragonskin 30–TPU 94A specimens with the 1.4 mm embedded patterns: (**a**) Pattern A-major; (**b**) Pattern A-minor; (**c**) Pattern B-major; (**d**) Pattern B-minor; (**e**) Pattern C.

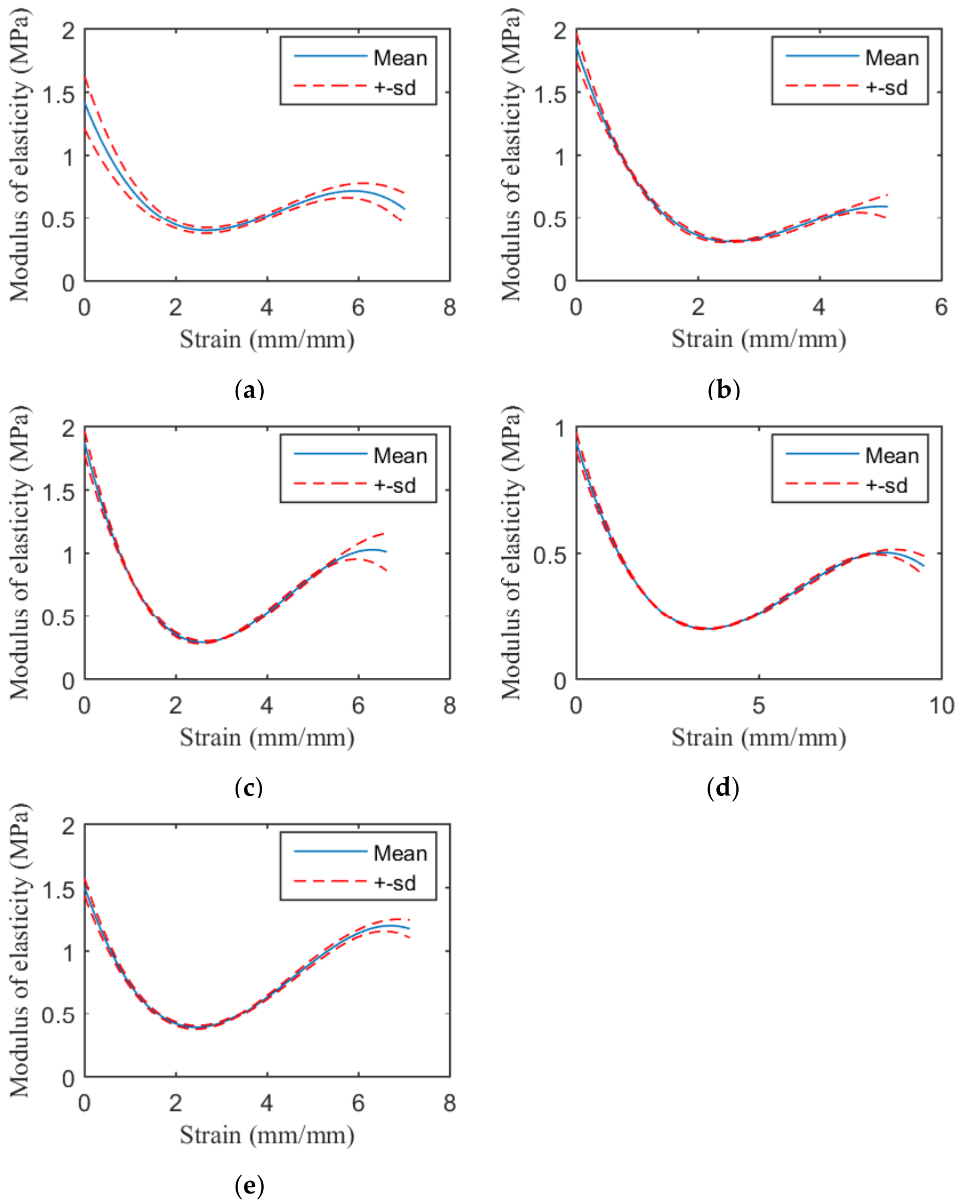

Figure 11. Modulus of elasticity of the TPU 94A specimens with the 0.7 mm embedded patterns: (**a**) Pattern A-major; (**b**) Pattern A-minor; (**c**) Pattern B-major; (**d**) Pattern B-minor; (**e**) Pattern C.

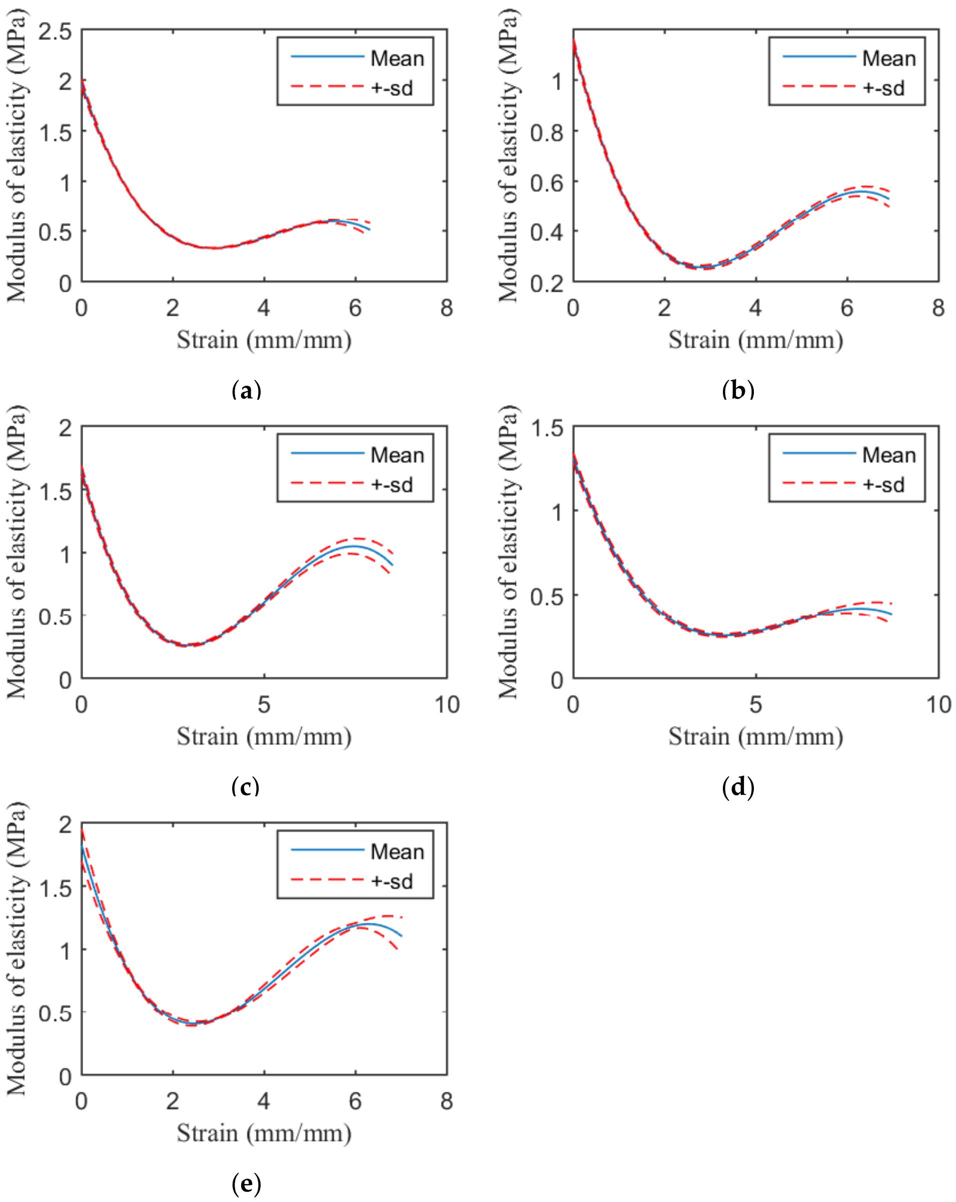

Figure 12. Modulus of elasticity of the TPU 94A specimens with the 1.4 mm patterns: (**a**) Pattern A-major; (**b**) Pattern A-minor; (**c**) Pattern B-major; (**d**) Pattern B-minor; (**e**) Pattern C.

Publisher's Note: MDPI stays neutral with regard to jurisdictional claims in published maps and institutional affiliations.

© 2020 by the authors. Submitted for possible open access publication under the terms and conditions of the Creative Commons Attribution (CC BY) license (http://creativecommons.org/licenses/by/4.0/).
